# M-Encapsulated Be_12_O_12_ Nano-Cage (M = K, Mn, or Cu) for CH_2_O Sensing Applications: A Theoretical Study

**DOI:** 10.3390/nano14010007

**Published:** 2023-12-19

**Authors:** Hatim Omar Al-Nadary, Khaled Mahmoud Eid, Heba Mohamed Badran, Hussein Youssef Ammar

**Affiliations:** 1 Physics Department, College of Science & Arts, Najran University, Najran 11001, Saudi Arabia; hoalnadary@nu.edu.sa; 2 Physics Department, Faculty of Education, Ain Shams University, Cairo 11566, Egypt; khaledmahmoud@edu.asu.edu.eg

**Keywords:** DFT and TD-DFT, beryllium oxide, formaldehyde, adsorption, sensor, alkali and transition metals

## Abstract

DFT and TD-DFT studies of B3LYP/6–31 g(d,p) with the D2 version of Grimme’s dispersion are used to examine the adsorption of a CH_2_O molecule on Be_12_O_12_ and MBe_12_O_12_ nano-cages (M = K, Mn, or Cu atom). The energy gap for Be_12_O_12_ was 8.210 eV, while the M encapsulation decreased its value to 0.685–1.568 eV, whereas the adsorption of the CH_2_O gas decreased the E_g_ values for Be_12_O_12_ and CuBe_12_O_12_ to 4.983 and 0.876 eV and increased its values for KBe_12_O_12_ and MnBe_12_O_12_ to 1.286 and 1.516 eV, respectively. The M encapsulation enhanced the chemical adsorption of CH_2_O gas with the surface of Be_12_O_12_. The UV-vis spectrum of the Be_12_O_12_ nano-cage was dramatically affected by the M encapsulation as well as the adsorption of the CH_2_O gas. In addition, the adsorption energies and the electrical sensitivity of the Be_12_O_12_ as well as the MBe_12_O_12_ nano-cages to CH_2_O gas could be manipulated with an external electric field. Our results may be fruitful for utilizing Be_12_O_12_ as well as MBe_12_O_12_ nano-cages as candidate materials for removing and sensing formaldehyde gas.

## 1. Introduction

Recently, the problem of pollution of the air, soil, and water has attracted the attention of many scientists. There have been several attempts to reduce pollution sources, whether by capturing the pollutants or detecting the contaminated materials. Formaldehyde (CH_2_O) is considered one of these pollutants. That is because of its diverse household uses in addition to its assorted uses in several industries [1,2,3,4,5,6,7,8]. CH_2_O gas has a pungent smell and no color [9]. Exposure to CH_2_O gas can cause voluminous hazards or even death to humans [10,11,12,13]. Accordingly, it is necessary to search for a material capable of removing the CH_2_O or that can be used as a CH_2_O sensor. Beryllium oxide (BeO) has some electronic features, making it a candidate material for this purpose. BeO is a semiconductor possessing an energy gap (E_g_) value of 8.21 eV [14]. The Be-O bond appears to be a shared behavior of ionic and covalent bonds [15]. Several experimental and theoretical efforts have been made to investigate BeO nano-structures, which have been synthesized in various forms such as nano-fibers [16] and nano-particles [17,18]. The geometrical and electrical characteristics of diverse nano-clusters of BeO have been inspected, and this has proven that a Be_12_O_12_ nano-cage possesses adequate stability [19]. Moreover, Be_12_O_12_ is a semiconductor with E_g_ = 7.41–8.29 eV [20,21,22,23,24,25]. Furthermore, metal oxide gas sensors have desirable properties such as a small size, lower cost, and extended lifetime [26]. Accordingly, Be_12_O_12_ nano-cages are employed for various uses such as a catalyst to convert methane to organic compounds [27]; a sensor for sulfur mustard [23], tabun, mercaptopyridine, formaldehyde, sulfur hydride, and sulfur dioxide [20,22,28,29]; and a hydrogen storage material [25]. The impact of Li, Na, and K deposition and encapsulation on the electronic and non-linear optical properties of Be_12_O_12_ has been studied [24,28]. It was found that the deposition and encapsulation of the alkali metals sharply decreased the E_g_ of the nano-cage to 1.10–1.56 eV and 0.68–0.69 eV, respectively. Additionally, it proved that the non-linear optical properties of Be_12_O_12_ nano-cages could be modified by the alkali metals. The UV-vis spectra for the Be_12_O_12_ nano-cage were predicted by Fallahi et al. [22] and Jouypazadeh et al. [23]. They found that the λ_max_ absorbance peak for a Be_12_O_12_ nano-cage is located at 140 to 150 nm. Kosar et al. [30] found that the encapsulation of Be_12_O_12_ with Be, Mg, and Ca atoms led to a red shift that was located at 505 nm, 781 nm, and 1136 nm, respectively.

Several efforts have been made to sense the CH_2_O molecules [31,32]. Diverse compounds such as MgO [33], BN [34,35,36,37], N-doped TiO_2_ [38], ZnO [4,39], transition metal-doped MoS_2_ [40], SnS [41], In_2_O_3_ [42], SnO_2_ [43], NiO [44], and BeO [20] have been employed as CH_2_O sensors.

According to previous studies [45,46,47,48,49,50,51,52], the existence of an external electric field (EF) impacts the geometric structure, substrate–adsorbate interaction, and electrical characteristics. Additionally, the electric field has a significant impact on key sensor properties such as the adsorption energy (E_ads_), sensitivity, and recovery time (τ). Our previous study [20] focused on studying the effect of the EF on the interaction characteristics of CH_2_O on a pristine Be_12_O_12_ nano-cage in different solvents. It was discovered that the magnitude and orientation of the EF can alter the sensing parameters of Be_12_O_12_ for the CH_2_O molecule.

According to our knowledge, there is a deficiency in the investigation of the impact of encapsulation of 3d metal atoms on the electrical, optical, and adsorption features of Be_12_O_12_. Moreover, no work has been interested in the effect of the existence of an EF on the adsorption features of CH_2_O molecules on metal-encapsulated Be_12_O_12_. Therefore, in this work, we sought to study the effect of an alkali metal (K) or a transition metal (Mn or Cu) as well as the effect of an EF on the adsorption characteristics of the CH_2_O molecule on a Be_12_O_12_ nano-cage.

## 2. Methods

**Figure 1:**
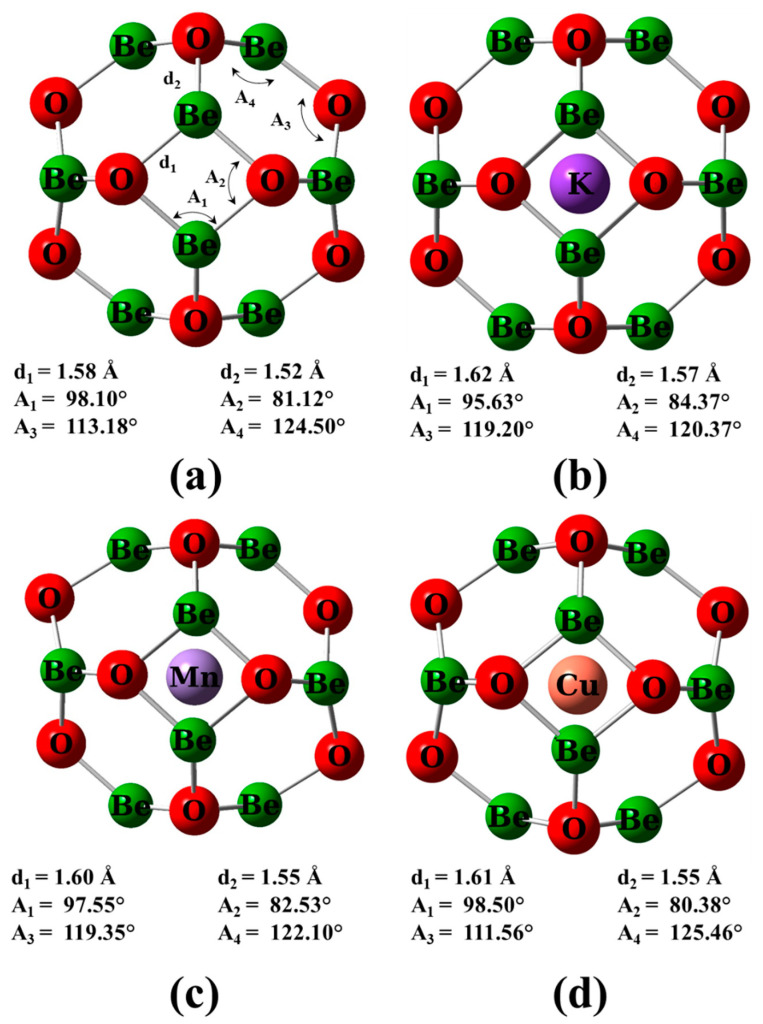
Optimized structures for (**a**) Be_12_O_12_, (**b**) KBe_12_O_12_, (**c**) MnBe_12_O_12_, and (**d**) CuBe_12_O_12_ nano-cages.

The BeO nano-cage was represented by 12 Be-O dimers. Then, as a trial to modify the properties of the beryllium oxide nano-cage (Be_12_O_12_), an M atom (M = K, Mn, or Cu) was encapsulated into the nano-cage to form MBe_12_O_12_ nano-cages (see Figure 1). DFT-D2 computations were performed to inspect the adsorption features of the CH_2_O molecule on the Be_12_O_12_ as well as MBe_12_O_12_ nano-cages. To estimate the optimized energetic geometrical structures and the electronic properties, the DFT-D2 computations were carried out by utilizing the hybrid functional B3LYP and 6–31 g(d,p) basis set [53]. The exchange functional B3 refers to Becke’s three parameters, while the correlation functional LYP refers to the correlation functional of Lee, Yang, and Parr [54]. The investigated structures were fully optimized under the following cutoff conditions for the total force on an atom (0.129 eV/Å), the root mean square of the force (0.086 eV/Å), the displacement ($5.29\times 10^{-3}Å$), and the root mean square of the displacement ($3.53\times 10^{-3}Å$).

D2 refers to Grimme’s dispersion [55,56], which takes into account the van der Waals interactions. The optimal multiplicity M (M = 2S + 1) was examined for the considered nano-cages, where S is the total spin. The dynamic stability for the considered nano-cages was checked by utilizing the vibrational frequencies, which were estimated by using the FT-IR spectra as well as a molecular dynamic simulation using the ADMP model. The UV-vis spectra were estimated for the considered structures by using the time-dependent DFT (TD-DFT) method. In TD-DFT calculations, an adequate number of excited states (n = 15) is estimated to cover all the probable transitions in the appropriate range (0–2000 nm).

The binding energy per atom (E_b_) for the investigated structures was assessed using Equation (1) [57].

(1) $$E_{b}=\frac{1}{n}\left(E_{cage}-\sum_{i=1}^{n}E_{i}\right)$$

Here, $E_{cage}$ is the optimized nano-cage total energy, $E_{i}$ is the single atom energy, and n is the number of the cage atoms.

The ionization potentials (IPs) for the considered cages were evaluated as shown below [5,58]:

(2) $$IP=E_{{cage}^{+}}-E_{cage}$$

where $E_{{cage}^{+}}$ is the energy of the cage after removing an electron while keeping the same geometry of the neutral cage. The Fermi level (E_F_), hardness (η), and electrophilicity (ω) were evaluated as shown in Equations (3)–(5), respectively [59,60]:

(3) $$E_{F}=\frac{1}{2}\left(E_{HOMO}+E_{LUMO}\right)$$

(4) $$\eta =\frac{1}{2}\left(E_{LUMO}-E_{HOMO}\right)\;$$

(5) $$\omega \approx E_{F}^{2}/2\eta \;$$

where $E_{HOMO}$ $E_{LUMO}$ are the energies of the highest occupied and the lowest unoccupied molecular orbitals, respectively. The adsorption energy (E_ads_) for CH_2_O on a nano-cage was calculated by Equation (6):

(6) $$E_{ads}=E_{CH_{2}O/cage}-\left(E_{cage}+E_{CH_{2}O}\right)$$

where $E_{CH_{2}O/cage}$, $E_{cage}\;$and $E_{CH_{2}O}$ are the energies for the $CH_{2}O/cage$ complex, the nano-cage and the CH_2_O molecule, respectively.

For the adsorption of multiple n molecules of CH_2_O gas, the adsorption energy per molecule (${\bar{E}}_{ads}$) was calculated by Equation (7):

(7) $${\bar{E}}_{ads}=\frac{1}{n}\left(E_{nCH_{2}O/cage}-\left(E_{cage}+nE_{CH_{2}O}\right)\right)$$

where $E_{nCH_{2}O/cage}\;$is the energy for the $nCH_{2}O/cage$ complex. A negative sign for the E_ads_ and ${\bar{E}}_{ads}$ indicates an exothermic reaction and more stable products.

The Gaussian 09 program was used to achieve all the calculations, whereas the Gauss View 5 was used for the visualization of the results [61]. The GaussSum 3.0 program estimated the partial density of states (PDOS) of the adsorbates–substrates [62]. The NBO version 3.1 measured the charges of atoms [63]. The Multiwfn 3.7 software package was employed to achieve the Quantum Theory of Atoms in Molecules (QTAIM) analysis [64].

## 3. Results and Discussion

### 3.1. Effect of Metal Encapsulation in Be_12_O_12_

In this section, the impact of doping on the geometrical, electrical, optical, and magnetic characteristics of the Be_12_O_12_ nano-cage was scrutinized. Therefore, full geometrical optimization was performed for the pristine and M-encapsulated Be_12_O_12_ nano-cage (M = K, Mn, or Cu). The optimized structures for Be_12_O_12_ as well as MBe_12_O_12_ are shown in Figure 1. The Be_12_O_12_ nano-cage comprises eight hexagonal and six tetragonal rings. Furthermore, the Be-O bonds are distinguished into two types. The first type, denoted by d_1_, is shared between hexagonal and tetragonal rings. The second type, denoted by d_2_, is shared between two hexagonal rings. It is found that d_1_ and d_2_ have bond lengths of 1.58 and 1.52 Å, respectively, for the Be_12_O_12_ nano-cage in good agreement with previous works [20,21,29,65]. Whereas the metal encapsulation into Be_12_O_12_ elongates d_1_ and d_2_ bonds to be 1.62 and 1.57 Å for KBe_12_O_12_, 1.60 and 1.55 Å for MnBe_12_O_12_, and 1.61 and 1.55 Å for CuBe_12_O_12_.

To investigate the stability of Be_12_O_12_ as well as MBe_12_O_12_ nano-cages, molecular dynamic (MD) simulations by the ADMP model as implemented in Gaussian 09 were performed. The MD simulations were performed at room temperature (300 K) for 500 fs. Figure S1, in the Supplementary Materials, illustrates the potential energy (PE) fluctuation against time besides the structure of Be_12_O_12_ as well as MBe_12_O_12_ at the end of the time. One can notice that the PE irrelevantly fluctuates, and unimportant distortion occurs for the cages. Therefore, the Be_12_O_12_ and MBe_12_O_12_ nano-cages have stable structures. Additionally, in Figure S2, in the Supplementary Materials, the disappearance of the imaginary frequencies for all investigated nano-cages declares that the optimized nano-cages are true minima on the potential energy surfaces [66,67,68,69]. This is in agreement with our previous work on the Be_12_O_12_ nano-cage [29].

One can judge the relative stability as well as relative reactivity of Be_12_O_12_ and MBe_12_O_12_ nano-cages in terms of the following: E_b_, HOMO–LUMO energy gap (E_g_), IP, E_F_, η, and ω. Their values are listed in Table 1. The E_b_ is calculated via Equation (1). The E_b_ value for Be_12_O_12_ is −5.630 eV, which is in agreement with Sajid et al. [21], while the M encapsulation decreases its value by 8.77%, 7.63%, and 4.01% for KBe_12_O_12_, MnBe_12_O_12_, and CuBe_12_O_12_, respectively. This indicates that the MBe_12_O_12_ nano-cages are lower in stability and consequently higher in reactivity than the Be_12_O_12_ nano-cage. Moreover, the E_g_ value for Be_12_O_12_ is 8.210 eV and it decreased to 0.685, 1.057, and 1.568 eV for KBe_12_O_12_, MnBe_12_O_12_, and CuBe_12_O_12_, respectively, which is in good agreement with previous studies [20,21,65].

Furthermore, the IP, E_F_, η, and ω values for Be_12_O_12_ are 10.135, −4.358, 4.105, and 2.314 eV, respectively. While the IP values decrease by 67.46%, 64.10%, and 48.54%, the E_F_ values lower to −1.786, −1.753, and −2.760 eV, and the η values decrease by 91.65%, 87.12%, and 80.90%, whereas the ω values rise by 101.18%, 25.56%, and 110.07% for KBe_12_O_12_, MnBe_12_O_12_, and CuBe_12_O_12_, respectively. It was stated [69,70,71,72] that the clusters distinguished by lower E_g_, IP, and η values and larger ω values are chemically less stable and more reactive. Thus, our results assert that the M-encapsulated Be_12_O_12_ nano-cages are chemically higher in reactivity than the Be_12_O_12_ nano-cage.

For more intuition, the NBO atomic charges were estimated. As listed in Table 1, for MBe_12_O_12_ nano-cages, the K, Mn, and Cu atoms own positive charges of 0.096, 0.237, and 0.108 e.

**Figure 2:**
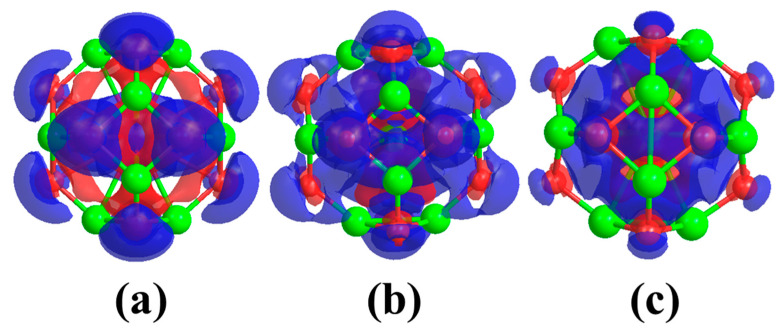
Charge density difference (Δρ) at 0.001 au isovalue for (**a**) KBe_12_O_12_ (**b**) MnBe_12_O_12_ (**c**) CuBe_12_O_12_ nano-cages. Red and blue colors refer to negative and positive Δρ values; $\Delta \rho =\rho_{{MBe}_{12}O_{12}}-(\rho_{{Be}_{12}O_{12}}+\rho_{M})$.

This indicates the occurrence of charge transfer from the M atoms to the rest of the beryllium oxide nano-cage. Despite the charge transfer that occurred, the charge distribution on the beryllium and oxygen atoms is still symmetric. Therefore, no obvious change in the magnitudes of the dipole moment values of the MBe_12_O_12_ nano-cages was observed. Figure 2 represents the charge density difference (Δρ) for MBe_12_O_12_ nano-cages.

Obviously, the M atom is enclosed by positive and negative values of Δρ, which indicates the occurrence of a donation-back donation of charges between the metal atom and the rest of the nano-cage atoms. In other words, there is a charge transfer from the M atom to the nano-cage and vice versa. Additionally, the electronic configuration was calculated for M atoms in a free state as well as in the MBe_12_O_12_ nano-cages and listed in Table 2. It is obvious that because of the interaction of the K, Mn, and Cu atoms with the rest of the KBe_12_O_12_, MnBe_12_O_12_, and CuBe_12_O_12_ nano-cages atoms, the 4s sublevel loses a charge of 0.54, 0.37, and 0.24 e, and the 4p sublevel gains 0.41, 0.41, and 0.48 e, respectively. Meanwhile, the 3d sublevel gains 0.23 and 0.76 e for KBe_12_O_12_ and MnBe_12_O_12_ nano-cages, respectively, and it loses 0.33 e for CuBe_12_O_12_ nano-cage. This emphasizes the donation-back donation mechanism of charges between the M atom and the rest of the nano-cage atoms.

**Figure 3:**
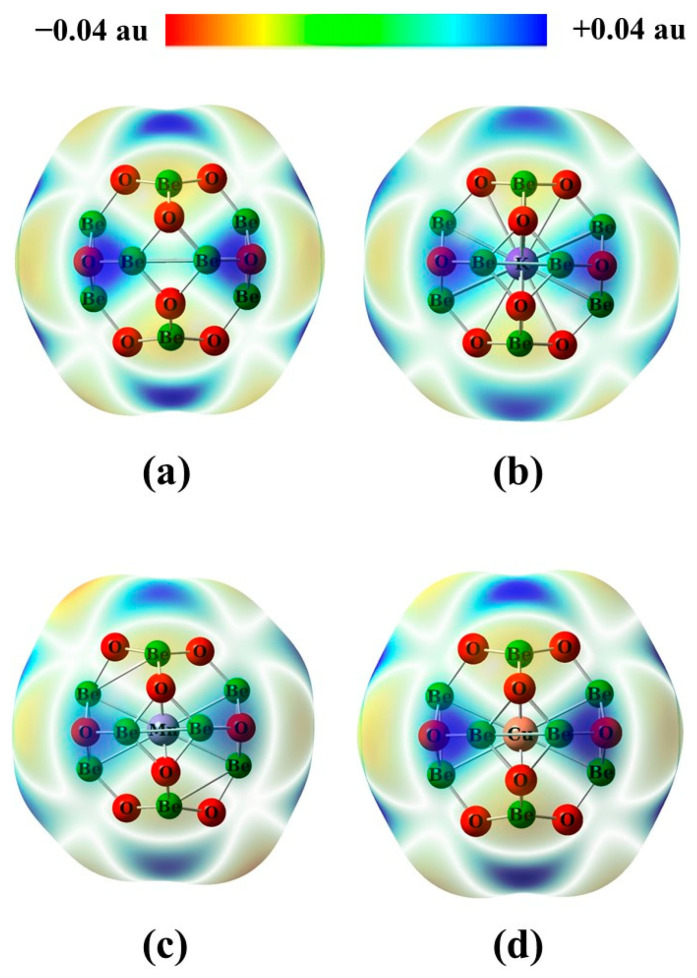
The molecular electrostatic potential maps (MESP) for (**a**) Be_12_O_12_, (**b**) KBe_12_O_12_, (**c**) MnBe_12_O_12_, and (**d**) CuBe_12_O_12_ nano-cages.

In Figure 3, the molecular electrostatic potential (MESP) for Be_12_O_12_ as well as MBe_12_O_12_ nano-cages was displayed. Figure 3a reveals that positive and negative electrostatic potentials surround the Be sites and the O sites, respectively, which is in good agreement with our previous work [20]. Figure 3b–d show that the K, Mn, or Cu encapsulation in the nano-cage has no obvious effect on the MESP distribution around the nano-cage. Therefore, the Be and O sites for Be_12_O_12_ as well as MBe_12_O_12_ nano-cages are expected to perform as electrophilic and nucleophilic sites, respectively.

**Figure 4:**
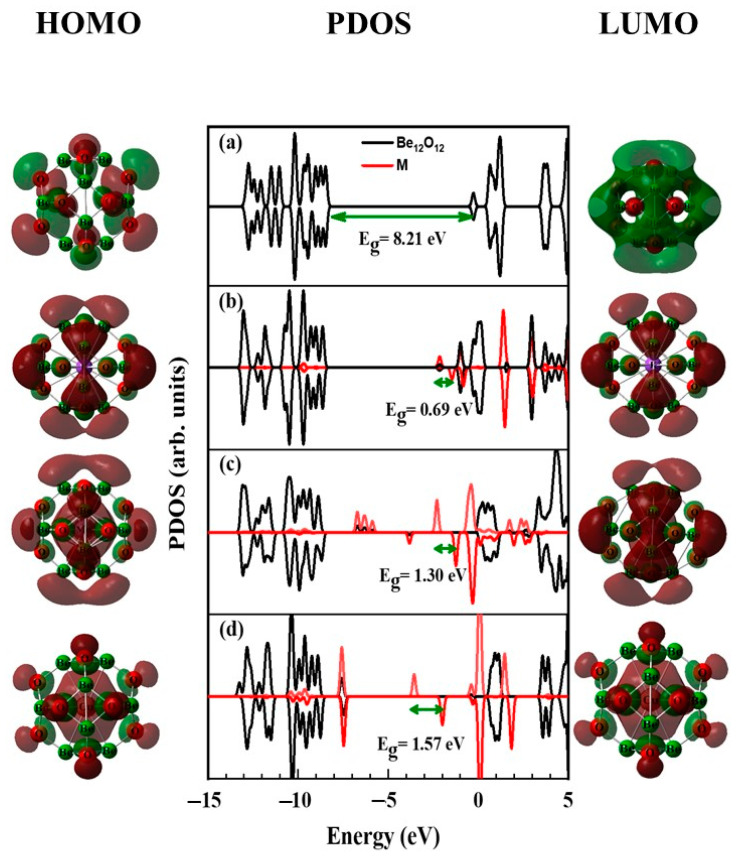
HOMO, PDOS, and LUMO for (**a**) Be_12_O_12_, (**b**) KBe_12_O_12_, (**c**) MnBe_12_O_12_, and (**d**) CuBe_12_O_12_ nano-cages. HOMO and LUMO surfaces are plotted at ± 0.02 isovalue. Brown and green colors refer to positive and negative isovalues, respectively.

Furthermore, the PDOS as well as the surfaces of the frontier orbitals (HOMO and LUMO) for Be_12_O_12_ and MBe_12_O_12_ nano-cages are depicted in Figure 4. As seen in Figure 4a the HOMO and LUMO of the Be_12_O_12_ nano-cage are located at −8.463 and −0.253 eV, respectively. Therefore, Be_12_O_12_ nano-cage is a wide-band gap semiconductor possessing an E_g_ value of 8.21 eV [14].

Additionally, the HOMO is commonly attributed to the 2p orbitals of the O atoms, whereas the LUMO is commonly attributed to the 2p orbitals of the Be atoms. Thus, the HOMO and LUMO are chiefly localized on the O and the Be sites, respectively. Figure 4b–d show that the presence of the K, Mn, and Cu atoms, respectively, causes noticeable changes in the states attributed to the Be_12_O_12_ nano-cage. Moreover, for KBe_12_O_12_, new donor and acceptor localized states appear at −2.14 and −1.45 eV, respectively. While for MnBe_12_O_12_, new donor localized states appear at −6.74, −6.62, −5.88, −3.84, and −2.28 eV, and new acceptor localized states appear at −0.98 and −0.38 eV. Whereas for CuBe_12_O_12_, new donor localized states appear at −7.58 and −3.54 and new acceptor localized states appear at −1.97, −0.36, and −0.02 eV. Consequently, the E_g_ values for KBe_12_O_12_, MnBe_12_O_12_, and CuBe_12_O_12_ are narrowed to 0.69, 1.30, and 1.57 eV, respectively. Furthermore, for MBe_12_O_12_ nano-cages, an overlap is observed between the occupied states of the K, Mn, and Cu atoms and the occupied states of the rest of the nano-cage atoms, which indicates an interaction among them has occurred. The electrical conductivity (σ) is governed by the E_g_ value as follows [73,74,75,76,77,78].

(8) $$\sigma \propto T^{3/2}exp(-\frac{E_{g}}{2kT})$$

where k is Boltzmann’s constant, and T is the temperature. Subsequently, the metal doping increases the σ value of the Be_12_O_12_ nano-cage.

**Figure 5:**
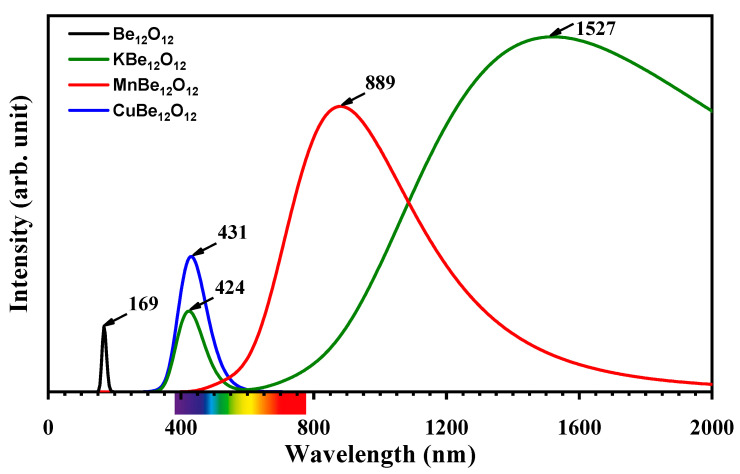
UV-vis spectra for Be_12_O_12_, KBe_12_O_12_, MnBe_12_O_12_, and CuBe_12_O_12_ nano-cages.

To scrutinize the influence of metal encapsulation on the optical properties of the Be_12_O_12_ nano-cage, the UV-vis spectra for the Be_12_O_12_, KBe_12_O_12_, MnBe_12_O_12_, and CuBe_12_O_12_ nano-cages were estimated via TD-DFT calculations and graphed in Figure 5. It is obvious the Be_12_O_12_ nano-cage has a λ_max_ absorbance peak in the UV region at 169 nm, which is consistent with previous studies [22,23]. Moreover, the KBe_12_O_12_ nano-cage has two absorbance peaks; the first peak is located in the visible region at 424 nm, and the second peak is located in the IR region at 1527 nm, while the MnBe_12_O_12_ nano-cage has a λ_max_ absorbance peak in the IR region at 889 nm. However, the CuBe_12_O_12_ nano-cage has a λ_max_ absorbance peak in the visible region at 431 nm. In other words, the metal encapsulation obviously affects the optical activity of the Be_12_O_12_ nano-cage. The Be_12_O_12_ nano-cage is an ultraviolet active compound, while the MBe_12_O_12_ nano-cages are active compounds in the visible and IR regions.

### 3.2. Adsorption of CH_2_O on M-Doped Be_12_O_12_

**Figure 6:**
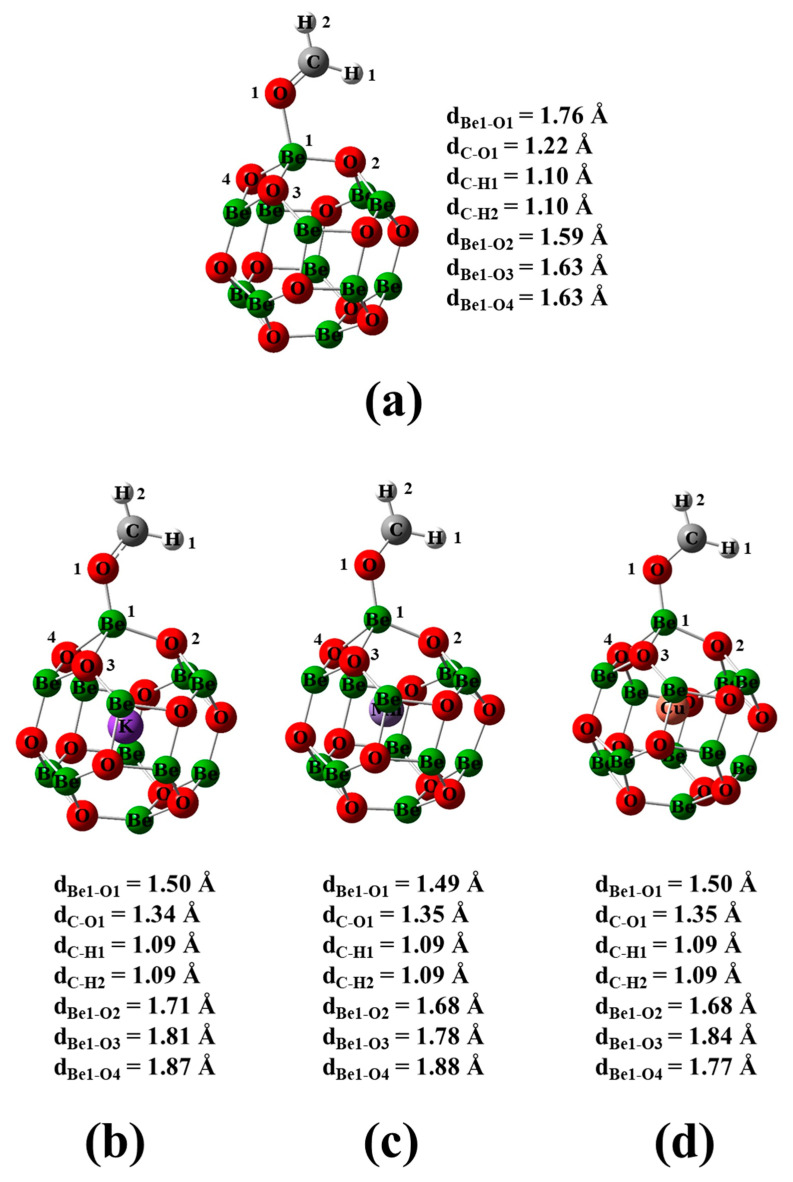
Optimized structures for (**a**) CH_2_O/Be_12_O_12,_ (**b**) CH_2_O/KBe_12_O_12,_ (**c**) CH_2_O/MnBe_12_O_12_, and (**d**) CH_2_O/CuBe_12_O_12_.

To examine the interaction characteristics of the CH_2_O molecule with the Be_12_O_12_ and MBe_12_O_12_ nano-cages, an optimization was carried out for the free CH_2_O, the bare nano-cages (Be_12_O_12_ and MBe_12_O_12_), and the CH_2_O/nano-cages (CH_2_O/Be_12_O_12_ and CH_2_O/MBe_12_O_12_) complexes. Our calculations show that the bond lengths of the C-O and C-H bonds for the CH_2_O molecule are 1.21 and 1.11 Å, respectively. The geometrical parameters for the bare Be_12_O_12_ and MBe_12_O_12_ nano-cages were discussed in the previous section. Additionally, in our previous work [20], it was found that negative and positive electrostatic potentials surround the O atom and CH_2_ group, respectively, of the CH_2_O molecule. While Figure 3 shows that positive and negative electrostatic potentials surround the Be and O sites, respectively, of the Be_12_O_12_ and MBe_12_O_12_ nano-cages. Therefore, the interaction of CH_2_O with the nano-cage is investigated in different orientations, as shown in Figure S3 in the Supplementary Materials. The optimization process for the suggested orientations shows that the CH_2_O molecule always interacts via its O head with the Be site of the nano-cage, as depicted in Figure 6.

One can see that the distance between the CH_2_O and the Be_12_O_12_ nano-cage (d_Be1-O1_ = 1.76 Å) is longer than that between the CH_2_O and the MBe_12_O_12_ nano-cage (d_Be1-O1_ in the range 1.49–1.50 Å). Moreover, the C-O bond length of the CH_2_O molecule is elongated by 0.83%, 10.74%, 11.57%, and 11.57% for the CH_2_O/Be_12_O_12_, CH_2_O/KBe_12_O_12_, CH_2_O/MnBe_12_O_12_, and CH_2_O/CuBe_12_O_12_, respectively. Furthermore, the CH_2_O adsorption elongates the bond lengths between the adsorbing site (Be1) and the neighboring oxygen sites (O2, O3, and O4); this elongation is higher for the CH_2_O/MBe_12_O_12_ complexes than that for the CH_2_O/Be_12_O_12_ complex. Table 3 lists the adsorption features of CH_2_O on MBe_12_O_12_ nano-cages.

One can notice that the CH_2_O adsorption on the Be_12_O_12_ as well as the MBe_12_O_12_ nano-cages is a chemisorption. Meanwhile, the E_ads_ values for the CH_2_O/MBe_12_O_12_ complexes are more negative than the E_ads_ value for the CH_2_O/Be_12_O_12_ complex. In other words, the presence of the M atom enhances the CH_2_O adsorption on the nano-cage. Thus, the Be_12_O_12_ as well as MBe_12_O_12_ nano-cages can be utilized as removal materials for formaldehyde gas, whereas the MBe_12_O_12_ nano-cages are more efficient than the pristine Be_12_O_12_ nano-cage for this purpose. Furthermore, it was found in previous studies that the E_ads_ of CH_2_O on the B_3_O_3_ monolayer [79], Ti-functionalized porphyrin-like C70 fullerenes [80], carbon nano-tube (CNT) [81], Pd-loaded CNT [81], BeO nano-tube [82], Zn_12_O_12_ nano-cage [4], and Al-deposited Zn_12_O_12_ nano-cage [4] are −0.402, −1.862, −0.106, −1.299, −1.088, −1.27, and −2.23 eV, respectively. Therefore, the present work shows that KBe_12_O_12_ and MnBe_12_O_12_ nano-cages as CH2O sorbent materials are more efficient than those mentioned in the previous studies. In addition, the CH_2_O adsorption decreases the E_g_ value for Be_12_O_12_ and CuBe_12_O_12_ by 39.31% and 44.13%, respectively, and increases its value for KBe_12_O_12_ and MnBe_12_O_12_ by 87.74% and 43.42%, respectively. For a more detailed explanation of the results, NBO atomic charge analysis, charge density difference (Δρ) analysis, QTAIM analysis, and PDOS analysis were performed. The NBO atomic charge analysis shows that for the CH_2_O/Be_12_O_12_ complex, the CH_2_O molecule earns a positive charge of 0.148 e due to a charge transfer from the CH_2_O molecule to the Be_12_O_12_ nano-cage. On the other side, for CH_2_O/MBe_12_O_12_ complexes, the CH_2_O molecule acquires negative charges of 0.703, 0.960, and 0.960 e. Furthermore, the positive charges for K, Mn, and Cu rose by 0.347, 0.333, and 0.470 e for CH_2_O/KBe_12_O_12_, CH_2_O/MnBe_12_O_12_, and CH_2_O/CuBe_12_O_12_, respectively; i.e., a charge transfer has occurred from the MBe_12_O_12_ nano-cages, mainly from the metal atom to the CH_2_O molecule. This may be owing to the lower IP value and consequently higher ability to donate electrons (higher basicity) for the MBe_12_O_12_ nano-cages than the Be_12_O_12_ nano-cages. Additionally, the electronic configurations of 2s and 2p orbitals for the O and C atoms of CH_2_O were estimated for the free CH_2_O, CH_2_O/Be_12_O_12,_ and CH_2_O/MBe_12_O_12_ complexes (see Table 4).

**Figure 7:**
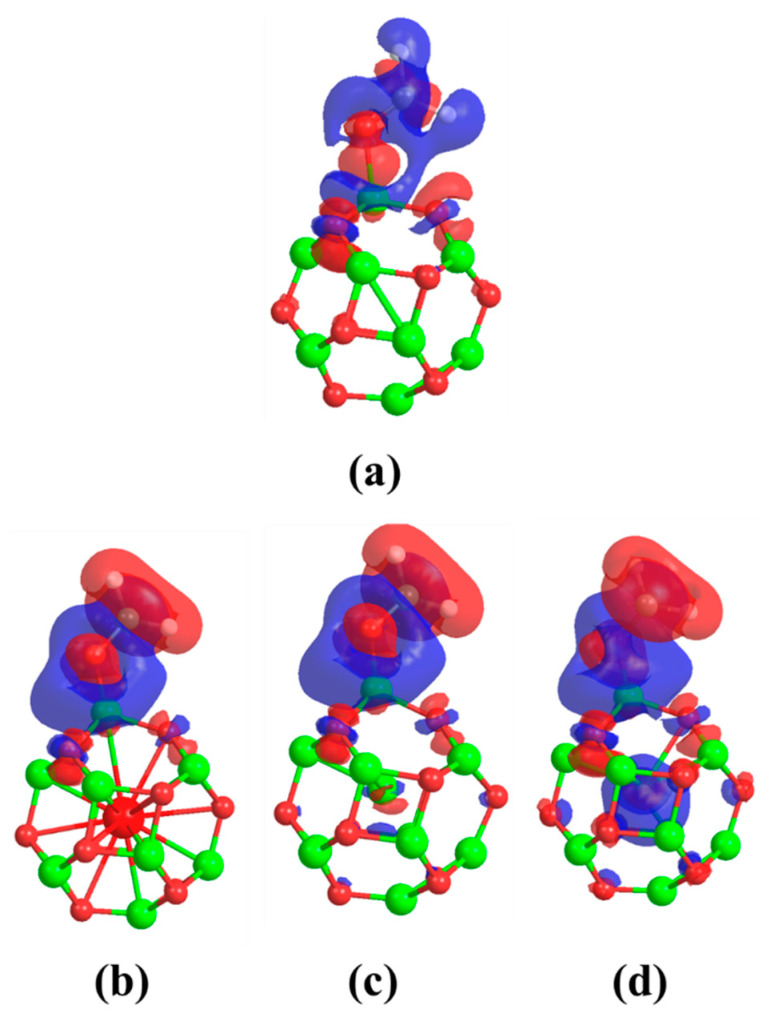
Charge density difference (Δρ) at 0.001 au isovalue for (**a**) CH_2_O/Be_12_O_12_, (**b**) CH_2_O/KBe_12_O_12_, (**c**) CH_2_O/MnBe_12_O_12_, and (**d**) CH_2_O/CuBe_12_O_12_ complexes. Red and blue colors refer to negative and positive Δρ values; $\Delta \rho =\rho_{CH_{2}O/{MBe}_{12}O_{12}}-(\rho_{M{Be}_{12}O_{12}}+\rho_{CH_{2}O})$.

It is clear that the interaction of the CH_2_O molecule with the Be_12_O_12_ as well as the MBe_12_O_12_ nano-cages is accompanied by losing electrons from the 2s and gaining electrons to the 2p orbitals of the oxygen atom. Whereas for the carbon atom, the 2s orbital has an insignificant change in its electronic configuration, while the 2p orbital loses electrons for the CH_2_O/Be_12_O_12_ complex and gains electrons for the CH_2_O/MBe_12_O_12_ complexes. This means a donation-back donation mechanism has happened between the CH_2_O molecule and the nano-cages. Figure 7 demonstrates the charge density difference (Δρ) for CH_2_O/Be_12_O_12_ as well as the CH_2_O/MBe_12_O_12_ complexes. One can see that the CH_2_O molecule for all the investigated complexes is surrounded by positive (blue color) and negative (red color) Δρ values. This means the CH_2_O molecule loses and gains charges, emphasizing the donation-back donation mechanism between the CH_2_O molecule and the nano-cages. It is worth noticing that the high dipole moment values for the CH_2_O/Be_12_O_12_ and the CH_2_O/MBe_12_O_12_ complexes in Table 3 refer to the charge redistribution between the CH_2_O molecule and the nano-cages.

For more insight into the nature of the CH_2_O adsorption on the Be_12_O_12_ as well as the MBe_12_O_12_ nano-cage, the QTAIM analysis was accomplished. Figure S4, in the Supplementary Materials, shows the bond critical points (BCP) of type (3,−1) for the CH_2_O/Be_12_O_12_ and the CH_2_O/MBe_12_O_12_ complexes. The topological parameters are stated in Table 5.

The kind of bond can be distinguished by the BCP parameters [83,84,85]. As reported, the ionic bond, weak hydrogen bond, and van der Waals interaction are characterized by $\nabla^{2}\rho$ > 0, H(r) > 0, −G(r)/V(r) > 1. Furthermore, the strong interaction is categorized by $\nabla^{2}\rho$ >10^−1^ au, and the weak interaction is categorized by $\nabla^{2}\rho$ < 10^−1^ au. Additionally, the partly covalent interaction is categorized by $\nabla^{2}\rho$ > 0 and H(r) < 0. Our results show that for the CH_2_O/Be_12_O_12_ complex, there are two BCPs; the first BCP is Be1-O1, which has a $\nabla^{2}\rho$ of 0.327 au, H(r) of 0.005 au, and −G(r)/V(r) ratio of 1.069, while the other BCP is O2-H1, which has a $\nabla^{2}\rho$ of 0.054 au, H(r) of 0.002 au, and −G(r)/V(r) ratio of 1.200. This indicates the formation of a pure ionic bond between the O1 atom of the CH_2_O molecule and the Be1 site of the Be_12_O_12_ nano-cage. Meanwhile, a weak hydrogen bond is found between the H1 atom of the CH_2_O molecule and the O2 site of the Be_12_O_12_ nano-cage. For CH_2_O/MBe_12_O_12_ complexes, only one BCP exists between the O1 atom of the CH_2_O molecule and the Be1 site of the MBe_12_O_12_ nano-cage. This BCP has $\nabla^{2}\rho$ values of 0.858, 0.879, and 0.867, −G(r)/V(r) ratios of 1.005, 1.000, and 1.005, and H(r) values of −0.002, −0.001, and −0.001 for CH_2_O/KBe_12_O_12_, CH_2_O/MnBe_12_O_12_, and CH_2_O/CuBe_12_O_12_ complexes, respectively. These results categorize the interaction as an ionic interaction with a partially covalent character. Therefore, one can say that the presence of the metal atom has an obvious role in altering the character of the interaction between the CH_2_O molecule and the nano-cages. Furthermore, the higher ρ values [80] for the CH_2_O/MBe_12_O_12_ complexes explain the higher adsorption of the CH_2_O molecule than the CH_2_O/Be_12_O_12_ complex.

**Figure 8:**
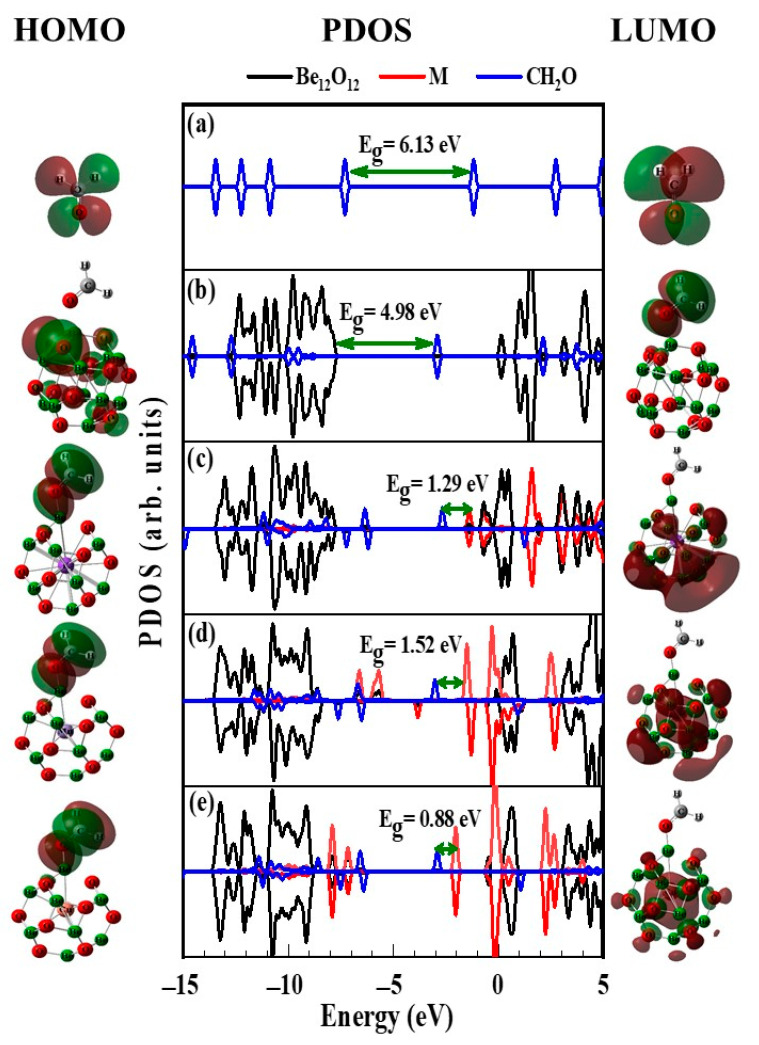
HOMO, PDOS, and LUMO for (**a**) CH_2_O, (**b**) CH_2_O/Be_12_O_12_, (**c**) CH_2_O/KBe_12_O_12_, (**d**) CH_2_O/MnBe_12_O_12_, and (**e**) CH_2_O/CuBe_12_O_12_ complexes.

Figure 8 depicts the HOMO and LUMO surfaces and the PDOS for the CH_2_O molecule, CH_2_O/Be_12_O_12_, and CH_2_O/MBe_12_O_12_ complexes. Figure 8a shows four occupied states of the CH_2_O molecule located at −7.30, −10.86, −12.24, and −13.44 eV. Looking at Figure 8b–e, obvious changes in the occupied states of the CH_2_O molecule indicate a strong reaction between the CH_2_O and the nano-cages. Regarding Figure 8b, one can see the HOMO of Be_12_O_12_ rises to −7.87 eV and an acceptor state is created at −2.89 eV; thus, the E_g_ value decreases from 8.21 to 4.98 eV. Looking at Figure 4b–d, it is obvious that there are occupied states located at −2.13, −2.28, and −3.54 eV, which are attributed to the metal atoms for KBe_12_O_12_, MnBe_12_O_12_, and CuBe_12_O_12_, respectively. As seen in Figure 8c–e, these states disappear for the complexes CH_2_O/KBe_12_O_12_, CH_2_O/MnBe_12_O_12_, and CH_2_O/CuBe_12_O_12_.

This confirms that the adsorption of the CH_2_O leads to a charge loss from the metal atom. Additionally, new occupied states assigned to CH_2_O appear at −2.67, −3.01, and −2.90 eV for CH_2_O/KBe_12_O_12_, CH_2_O/MnBe_12_O_12_, and CH_2_O/CuBe_12_O_12_, respectively. This confirms that the adsorption of the CH_2_O leads to a charge transfer from the MBe_12_O_12_ to the CH_2_O molecule. Furthermore, the adsorption of the CH_2_O molecule causes a shift for LUMO states from −1.44, −1.22, and −1.98 eV to −1.38, −1.49, and −2.02 eV for KBe_12_O_12_, MnBe_12_O_12_, and CuBe_12_O_12_, respectively. Accordingly, the E_g_ values increase from 0.69 and 1.06 eV to 1.29 and 1.52 eV for KBe_12_O_12_ and MnBe_12_O_12_ nano-cages, respectively, while E_g_ value decreases from 1.57 to 0.88 eV for CuBe_12_O_12_ nano-cage. Consequently, due to the CH_2_O adsorption and Equation (8), the electrical conductivity is increased for the Be_12_O_12_ and CuBe_12_O_12_, whereas it is decreased for the KBe_12_O_12_ and MnBe_12_O_12_. Therefore, the Be_12_O_12_ and CuBe_12_O_12_ nano-cages can be utilized as electrical sensors for the CH_2_O molecule. In addition, the essential sensing factor, the recovery time (τ), depends on the E_ads_ as in the following equation [20,86,87].

(9) $$\tau =\nu_{ο}^{-1}\exp(-\frac{E_{ads}}{kT})$$

where $\nu_{ο}$ is the attempt frequency, k is Boltzmann’s constant, and T is the temperature. Therefore, the τ value is in the following trend: MnBe_12_O_12_ > KBe_12_O_12_ > CuBe_12_O_12_ >Be_12_O_12_.

### 3.3. Effect of EF

**Figure 9:**
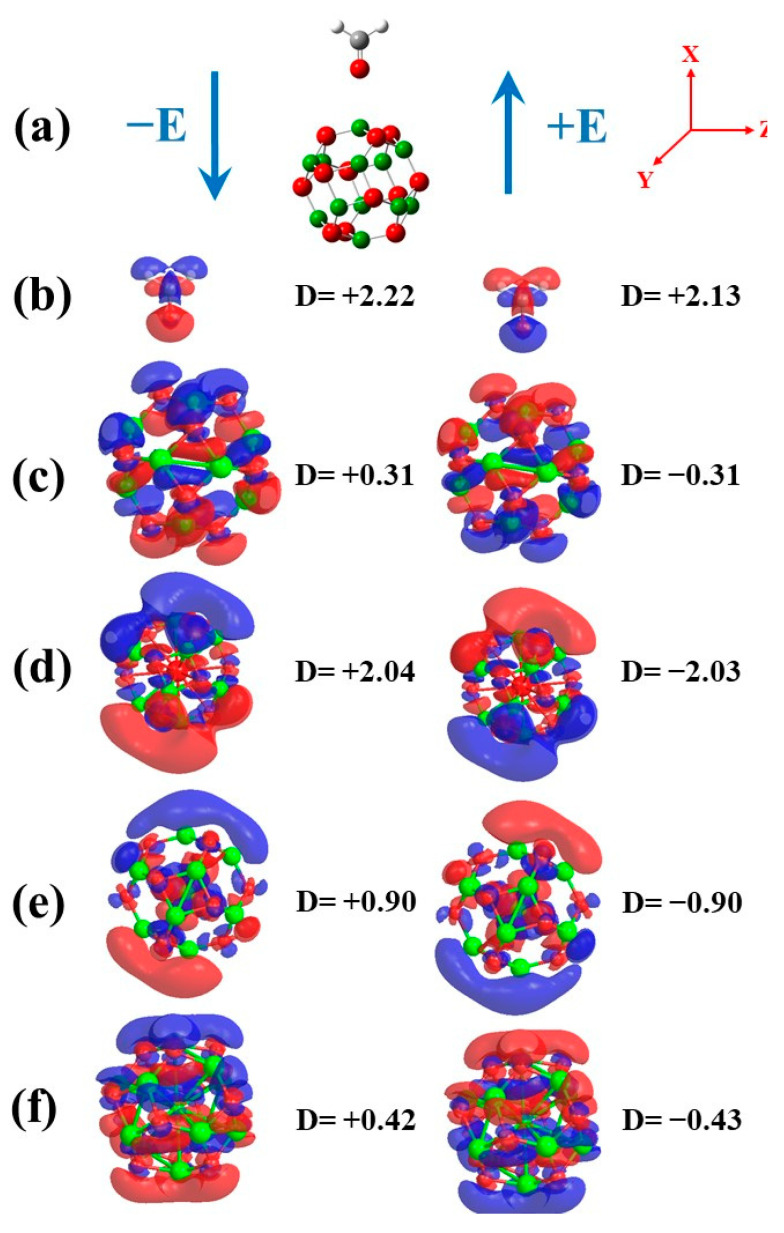
(**a**) Electric field direction relative to the nano-cage, and the charge density difference (Δρ) isovalue surfaces at 0.00005 au for EF values of −514 and +514 kV/mm for (**b**) CH_2_O, (**c**) Be_12_O_12_, (**d**) KBe_12_O_12_, (**e**) MnBe_12_O_12_, and (**f**) KBe_12_O_12_. $\Delta \rho =\rho_{\;E=\pm 514}-\rho_{\;E=0}$, X-component of dipole moment (D_x_) in Debye. Red and blue colors represent negative and positive Δρ values, respectively.

In the present section, the influence of EF on the electronic properties of the CH_2_O molecule, Be_12_O_12_ as well as MBe_12_O_12_ nano-cages, and the CH_2_O/Be_12_O_12_ as well as CH_2_O/MBe_12_O_12_ complexes was examined. The EF was considered in the range of −514 to +514 kV/mm with a step of 102.8 kV/mm. All the investigated structures are fully optimized for each EF value. The direction of the EF relative to the investigated structures is sketched in Figure 9. The influence of the EF on the dipole moment for the CH_2_O molecule and the Be_12_O_12_ nano-cage, as well as the MBe_12_O_12_ nano-cages, was investigated. Because the EF was applied in the X-axis direction, the change in the dipole moment was only evident in its x-component.

**Figure 10:**
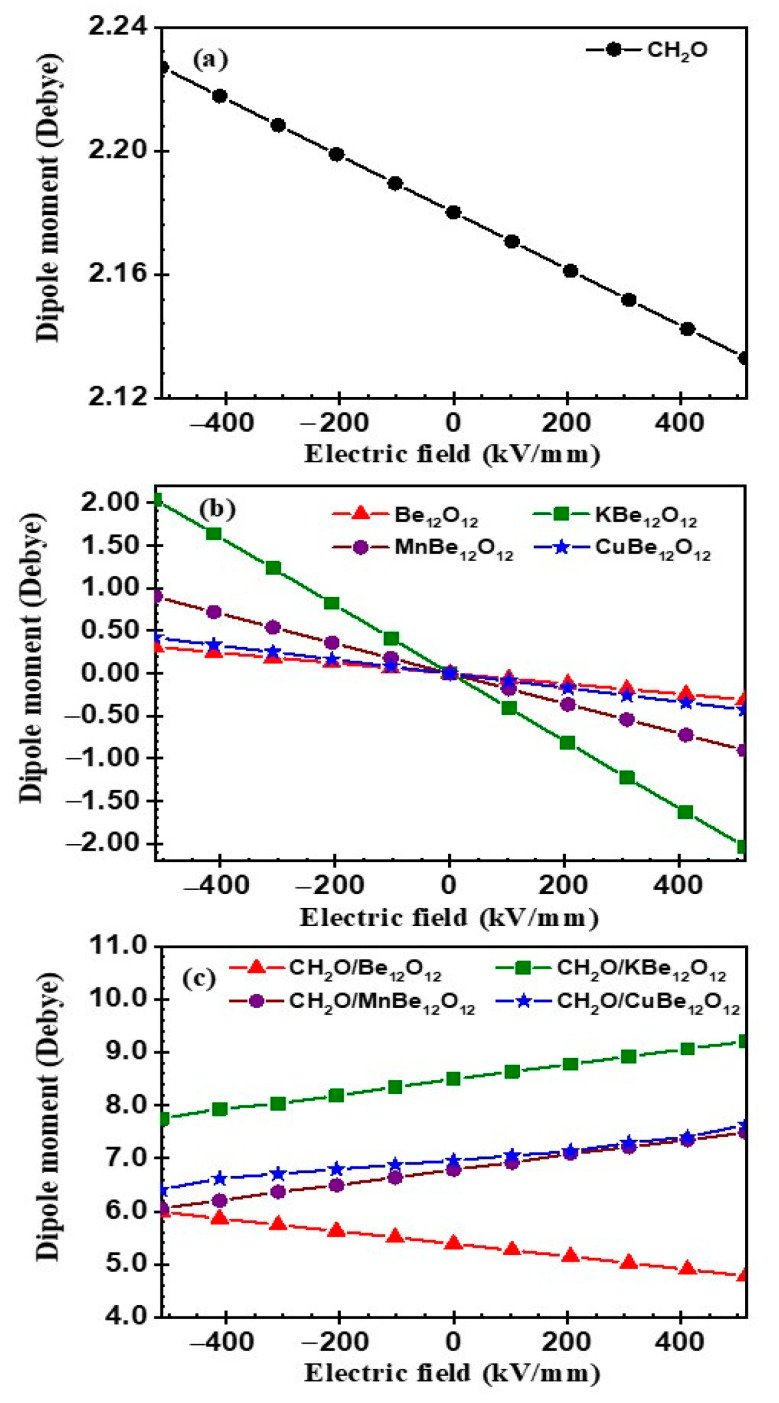
Dipole moment vs. electric field at different mediums for (**a**) CH_2_O, (**b**) Be_12_O_12_ and MBe_12_O_12_ substrates, and (**c**) CH_2_O/Be_12_O_12_ and CH_2_O/MBe_12_O_12_ complexes.

Therefore, the x-component only of the dipole moment was considered. Figure 10a shows the dipole moment for the CH_2_O molecule versus EF. It is clear that with the EF varying from −514 to +514 kV/mm, the dipole moment of the CH_2_O molecule decreases. The dipole moments versus EF for the Be_12_O_12_ and MBe_12_O_12_ nano-cages are illustrated in Figure 10b.

It is seen that with the varying of the EF from −514 to +514 kV/mm, the dipole moment of the investigated nano-cages decreases from 0.311, 2.041, 0.901, and 0.420 Debye to −0.311, −2.032, −0.903, and −0.427 Debye for Be_12_O_12_, KBe_12_O_12_, MnBe_12_O_12_, and CuBe_12_O_12_, respectively. It is worth noticing that the polarity of the dipole moment is inverted as the EF direction is inverted. Additionally, the rate of change in the dipole moment with the EF is in this trend: KBe_12_O_12_ > MnBe_12_O_12_> CuBe_12_O_12_> Be_12_O_12_. These results manifest that the EF affects the charge distribution for the CH_2_O molecule, Be_12_O_12_, and MBe_12_O_12_ nano-cages. This is emphasized by Figure 10b–f, which demonstrates the charge density difference (Δρ) for the CH_2_O molecule, Be_12_O_12_, and MBe_12_O_12_ nano-cages for EF values of −514 and +514 kV/mm. The dipole moment of a molecule plays an important role in its reactivity with the surrounding medium [88]. Therefore, it is expected that the EF would have an obvious impact on the CH_2_O adsorption on the Be_12_O_12_ as well as MBe_12_O_12_nano-cages.

**Figure 11:**
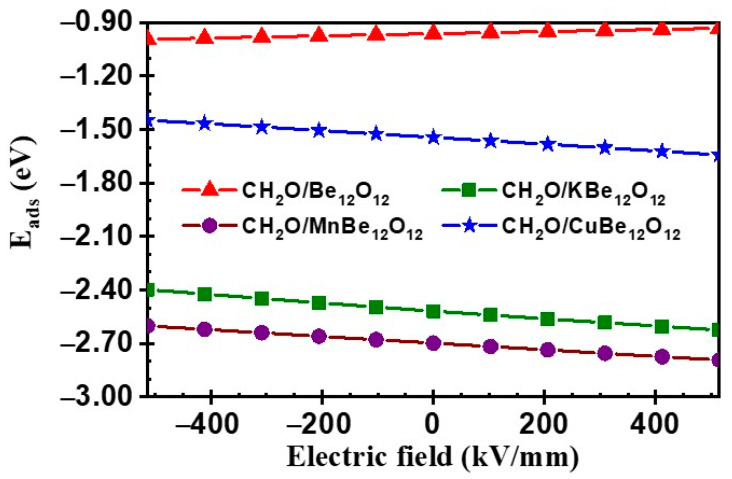
Adsorption energies (E_ads_) for CH_2_O/Be_12_O_12_ and CH_2_O/MBe_12_O_12_ complexes.

The manipulation of the EF on the CH_2_O adsorption on the Be_12_O_12_ and MBe_12_O_12_ nano-cages has been completed under the same criteria mentioned above. The E_ads_ values for the CH_2_O/Be_12_O_12_ and CH_2_O/MBe_12_O_12_ complexes are estimated and plotted against the EF in Figure 11.

**Figure 12:**
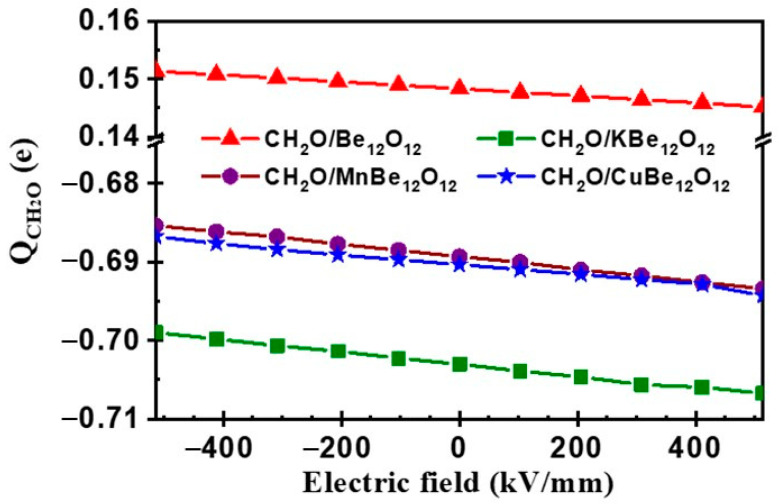
NBO charges of CH_2_O molecule ($Q_{CH_{2}O}$) vs. electric field for CH_2_O/Be_12_O_12_ and CH_2_O/MBe_12_O_12_ complexes.

It is seen that by increasing the negative EF, the value of E_ads_ is gradually enhanced for the CH_2_O/Be_12_O_12_ complex up to 3.4% at EF = −514 kV/mm with respect to their value at zero EF. Meanwhile, the values of E_ads_ are gradually inhibited for CH_2_O/KBe_12_O_12_, CH_2_O/MnBe_12_O_12_, and CH_2_O/CuBe_12_O_12_ complexes up to 4.8%, 3.6%, and 6.2%, respectively, at EF = −514 kV/mm with respect to its value at zero EF. On the other side, by increasing the positive EF, the value of E_ads_ is gradually inhibited for the CH_2_O/Be_12_O_12_ complex up to 3.1% at EF = +514 kV/mm with respect to its value at zero EF. Meanwhile, the values of E_ads_ are gradually enhanced for CH_2_O/KBe_12_O_12_, CH_2_O/MnBe_12_O_12_, and CH_2_O/CuBe_12_O_12_ complexes up to 4.2%, 3.5%, and 6.4%, respectively, at EF = +514 kV/mm with respect to their value at zero EF. Therefore, the E_ads_ values for CH_2_O/Be_12_O_12_ and CH_2_O/MBe_12_O_12_ complexes are dominated by either the value or direction of the EF. These results could be explained in terms of the mechanism of CH_2_O interaction with the nano-cage. For the CH_2_O/Be_12_O_12_ complex, as mentioned before, there is a charge transfer from the CH_2_O molecule to the Be_12_O_12_ nano-cage. Looking at Figure 9b,c, one can see that the negative EF induces a negative Δρ value on the oxygen head of the CH_2_O molecule and a positive Δρ value on the side of the Be_12_O_12_ nano-cage facing the CH_2_O molecule. This in turn encourages the charge transfer and, consequently, enhances the E_ads_ value. In contrast, the positive electric field induces a positive Δρ value on the oxygen head of the CH_2_O molecule and a negative Δρ value on the side of the Be_12_O_12_ nano-cage facing the CH_2_O molecule. This discourages the charge transfer and, consequently, inhibits the E_ads_ value. This could be confirmed by Figure 12, which shows the NBO charges of the CH_2_O molecule ($Q_{CH_{2}O}$) vs. electric field. It is clear that the positive $Q_{CH_{2}O}$ for the CH_2_O/Be_12_O_12_ complex increases as the negative EF increases and decreases as the positive EF increases.

Thus, the E_ads_ value is enhanced by increasing the negative EF and inhibited by increasing the positive EF (see Figure 11). On the other side, for the CH_2_O/MBe_12_O_12_ complexes, the interaction has occurred due to the charge transfer from the MBe_12_O_12_ nano-cage to the CH_2_O molecule. Looking at Figure 9d–f, it is clear that for negative EF values, the induced positive Δρ value on the side of the MBe_12_O_12_ nano-cage facing the CH_2_O molecule inhibits this charge transfer and consequently inhibits the E_ads_ values. Whereas for the positive EF values, the induced negative Δρ value on the side of the MBe_12_O_12_ nano-cage facing the CH_2_O molecule encourages the charge transfer and thus enhances the E_ads_ values. This can be confirmed by Figure 12, where the negative $Q_{CH_{2}O}$ for CH_2_O/MBe_12_O_12_ complexes decreases as the negative EF increases and decreases as the positive EF increases. Thus, the E_ads_ value is inhibited as the negative EF increases and enhanced as the positive EF increases (see Figure 11).

**Figure 13:**
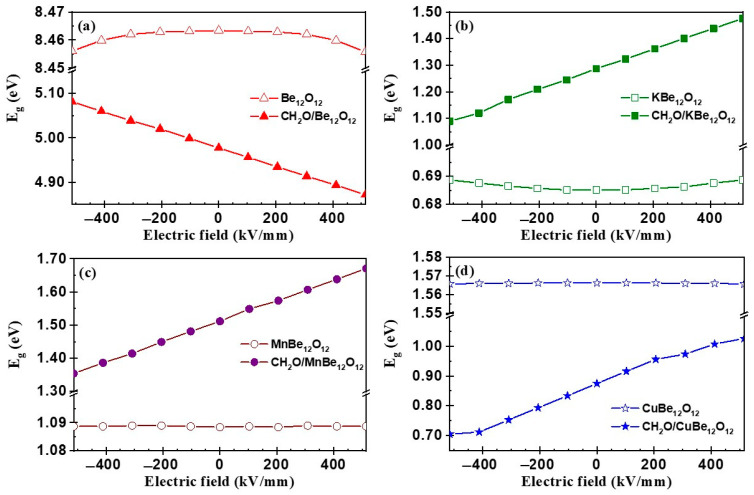
HOMO–LUMO energy gap (E_g_) vs. electric field for (**a**) Be_12_O_12_ and CH_2_O/Be_12_O_12_, (**b**) KBe_12_O_12_ and KCH_2_O/Be_12_O_12_, (**c**) MnBe_12_O_12_ and CH_2_O/MnBe_12_O_12_, and (**d**) CuBe_12_O_12_ and CH_2_O/CuBe_12_O_12_.

Figure 13 represents the impact of the EF on the E_g_ for the Be_12_O_12_ and MBe_12_O_12_ nano-cages as well as the CH_2_O/Be_12_O_12_ and CH_2_O/MBe_12_O_12_ complexes. It is clear that the EF has a negligible impact on the E_g_ values of the Be_12_O_12_ and MBe_12_O_12_ nano-cages. Figure 13a declares that the E_g_ value for the CH_2_O/Be_12_O_12_ complex increases as the negative EF increases and decreases as the positive EF increases. On the other side, Figure 13b–d show that the E_g_ values for the CH_2_O/KBe_12_O_12_, CH_2_O/MnBe_12_O_12_, CH_2_O/CuBe_12_O_12_ complexes increase as the negative EF decreases and the positive EF increases. Therefore, according to Equation (8), the electric conductivity (σ) for the CH_2_O/Be_12_O_12_ and CH_2_O/MBe_12_O_12_ complexes can be controlled by the EF. Here, σ for the CH_2_O/Be_12_O_12_ complexes will increase by increasing the positive EF, while for the CH_2_O/MBe_12_O_12_ complexes, σ will increase by increasing the negative EF.

**Figure 14:**
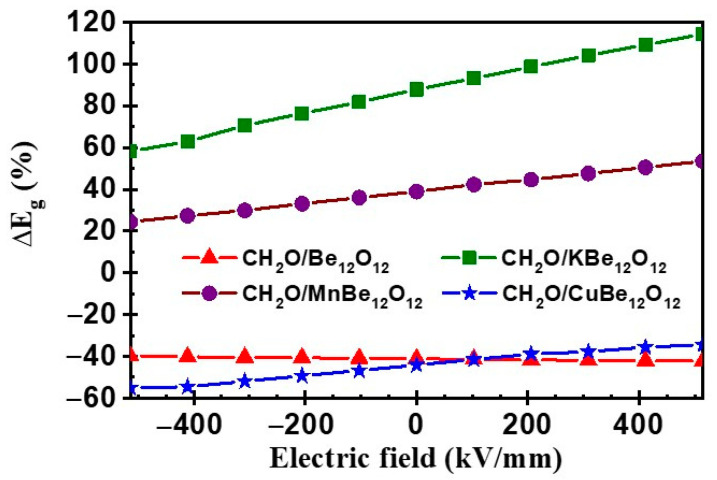
The change percentage for HOMO–LUMO energy gap (ΔE_g_) vs. electric field.

To investigate the electrical sensitivity dependence on the EF, the percentage of the variance of E_g_ (ΔE_g_) by the adsorption for CH_2_O/Be_12_O_12_ and CH_2_O/MBe_12_O_12_ complexes versus the EF is estimated by Equation (10) and represented in Figure 14.

(10) $$\Delta E_{g}=\frac{E_{g}({CH}_{2}O/nano-cage)-E_{g}(nano-cage)}{E_{g}(nano-cage)}\times 100$$

ΔE_g_ values at EF = 0 were −41.2%, 87.8%, 38.9%, and −44.1% for CH_2_O/Be_12_O_12_, CH_2_O/KBe_12_O_12_, CH_2_O/MnBe_12_O_12_, and CH_2_O/CuBe_12_O_12_ complexes, respectively. It is clear that the variance in value and the direction of the EF has a negligible effect on ΔE_g_ for the CH_2_O/Be_12_O_12_ complex. Whereas for the CH_2_O/KBe_12_O_12_, CH_2_O/MnBe_12_O_12_, and CH_2_O/CuBe_12_O_12_ complexes, as the negative EF increases, the ΔE_g_ values are lowered, reaching 58.2%, 24.4%, and −55.0%, while as the positive EF increases, the ΔE_g_ values are rising, reaching 114.2%, 53.4%, and −34.4%, respectively. Therefore, the electrical sensitivity of Be_12_O_12_ to the adsorption of CH_2_O molecules is independent of the EF, while the electrical sensitivity of MBe_12_O_12_ depends on the EF.

### 3.4. UV-vis Spectra Investigation

**Figure 15:**
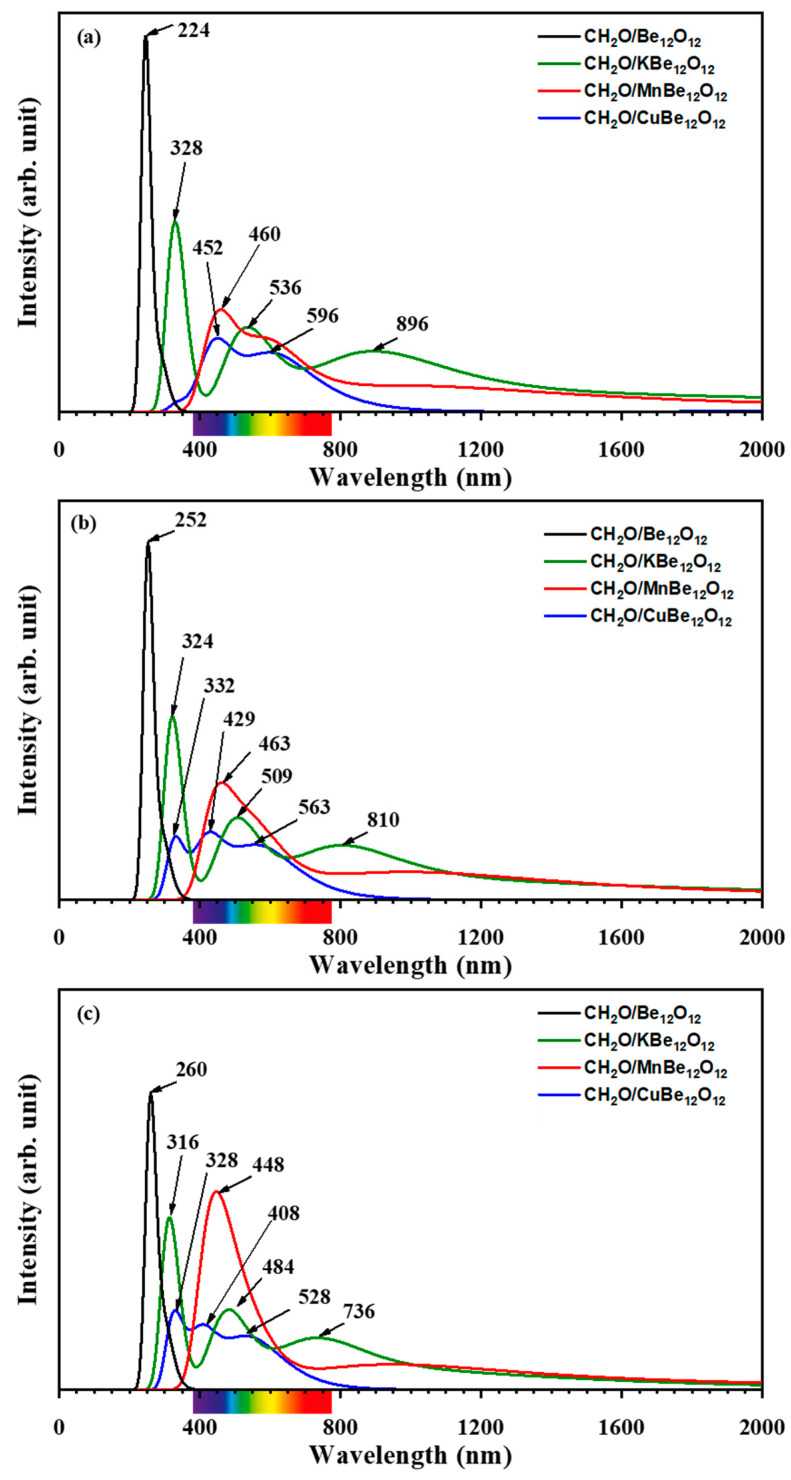
UV-vis spectra for CH_2_O/Be_12_O_12_, CH_2_O/KBe_12_O_12_, CH_2_O/MnBe_12_O_12_, and CH_2_O/CuBe_12_O_12_ complexes for EF values of (**a**) −514 kV/mm, (**b**) 0 kV/mm, and (**c**) +514 kV/mm, respectively.

Herein, the effect of the adsorption of the CH_2_O molecule as well as the EF on the UV-vis spectra of the examined nano-cages was considered. The UV-vis spectra for the CH_2_O/Be_12_O_12_ and CH_2_O/MBe_12_O_12_ complexes are predicted for EF values of −514, 0, and +514 kV/mm, and these are displayed in Figure 15a–c, respectively. It is worth noting that the effect of the EF on the UV-vis spectra of bare nano-cages is studied, and it is found that the EF has no noticeable effect. It is clear that at EF values of −514, 0, and +514 kV/mm, the CH_2_O/Be_12_O_12_ complex has a λ_max_ absorbance peak in the UV region at 224, 252, and 260 nm, respectively. Comparing these results with Figure 5, one can observe that the adsorption of CH_2_O causes a red shift for the UV-vis spectrum of the Be_12_O_12_. The negative EF value decreases the red shift value of λ_max_, while the positive EF increases it. Furthermore, at zero EF, the CH_2_O/KBe_12_O_12_ exhibits three peaks located at 324 nm, 509 nm, and 810 nm in the UV, visible, and IR regions of the spectra, respectively. The presence of the EF shifts these peaks to 328, 536, and 896 nm at negative EF and to 316, 484, and 736 nm at positive EF. When compared with Figure 5, the Kbe_12_O_12_ nano-cage has a peak in the visible region at 424 nm, whereas the adsorption of CH_2_O induces a red shift in this peak to 536, 509, and 484 for EF values of −514, 0, and +514 kV/mm, respectively. Moreover, at EF values of −514, 0, and +514 kV/mm, the CH_2_O/MnBe_12_O_12_ exhibits one prominent peak in the visible region of the spectra, which is located at 460, 463, and 448 nm, respectively. In comparison with Figure 5, it is evident that the MnBe_12_O_12_ nano-cage has one peak in the IR region (889 nm), while the adsorption of CH_2_O causes a blue shift for λ_max_ to the visible region. Additionally, Figure 5 shows that the CuBe_12_O_12_ nano-cage is optically active in the visible region with a λ_max_ value of 431 nm. Meanwhile, the CH_2_O/CuBe_12_O_12_ has peaks in the visible region at 452 and 596 nm at an EF value of −514 kV/mm, 332, 429, and 563 nm at an EF value of 0 kV/mm, and 328, 408 and 528 nm at an EF value of +514 kV/mm. From the above discussion, one can summarize the following: (i) the adsorption of a CH_2_O molecule affects the UV-vis spectra of Be_12_O_12_ as well as the MBe_12_O_12_. (ii) The EF has no effect on the UV-vis spectra of Be_12_O_12_ as well as the MBe_12_O_12_. (iii) The EF has an obvious effect on the UV-vis spectra of CH_2_O/Be_12_O_12_ as well as CH_2_O/MBe_12_O_12_. In other words, the adsorption of the CH_2_O molecule causes changes in the colors of the MBe_12_O_12_ nano-cages; consequently, they could be employed as bare-eye sensors for the CH_2_O molecule.

### 3.5. Effect of Concentration

**Figure 16:**
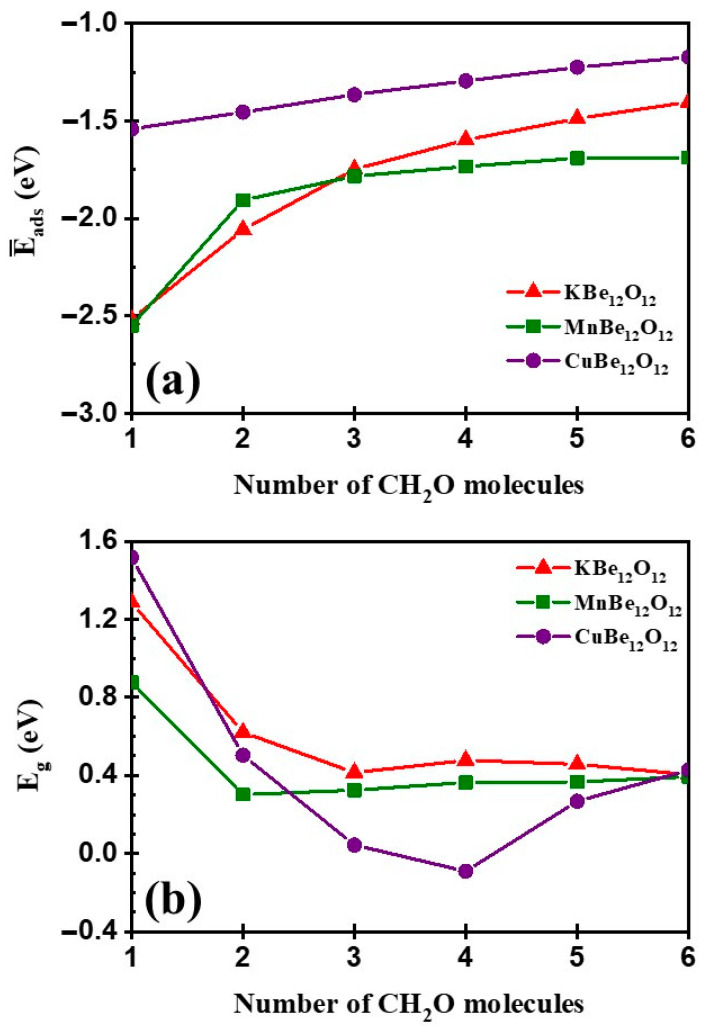
(**a**) $\bar{E}$_ads_ and (**b**) E_g_ against the number of adsorbed CH_2_O molecules.

This section concerns the impact of CH_2_O concentration on the adsorption characteristics. The adsorption of n molecules (n = 1–6) on the surface of the MBe_12_O_12_ was investigated to form nCH_2_O/MBe_12_O_12_ complexes. The nCH_2_O/MBe_12_O_12_ complexes were fully optimized. The adsorption energies per molecule (${\bar{E}}_{ads}$) were estimated by Equation (7) and graphed in Figure 16a. It is seen that as n increases, the negative value of the ${\bar{E}}_{ads}$ decreases. Nevertheless, the ${\bar{E}}_{ads}$ values remain within the bounds of chemical adsorption for all n values.

In Figure 16b, the relationship between E_g_ and n is illustrated. It is observed that for KBe_12_O_12_ and MnBe_12_O_12_, as n increases up to n = 3 and 2, respectively, the E_g_ decreases after which there is no significant change. Conversely, for CuBe_12_O_12_, the E_g_ decreases as n increases, reaching a minimum at n = 4, and then it rises again. According to Equations (8) and (10), it is noted that for all the investigated nano-cages, the electrical conductivity (σ) and the electrical sensitivity at all values of n > 1 are higher than their values at n = 1. Furthermore, the highest electrical conductivity (σ) and electrical sensitivity are recorded for CuBe_12_O_12_ at n = 4.

## 4. Conclusions

This work is a DFT and TD-DFT study that investigates the capability of the M atom-encapsulated Be_12_O_12_ nano-cage to capture or sense CH_2_O gas; M = K, Mn, or Cu. The molecular dynamic simulations and the frequency calculations assert that the Be_12_O_12_ and MBe_12_O_12_ nano-cages are stable structures. In contrast, the values of the binding energies per atom (E_b_), the ionization potential (IP), the hardness (η), and the electrophilicity (ω) prove that the M-encapsulated Be_12_O_12_ is chemically more reactive than the Be_12_O_12_ cage. In addition, the encapsulation of the M atom into the Be_12_O_12_ nano-cage increases its basicity, narrows its energy gap, and alerts its optical activity from an ultraviolet active compound into active compounds in the visible and IR regions.

Moreover, the calculated adsorption energies confirm that the CH_2_O adsorption on the Be_12_O_12_ as well as the MBe_12_O_12_ is chemisorption, while the presence of the M atom improves the adsorption of the CH_2_O molecule on the nano-cage. Moreover, the QTAIM analysis confirms that the presence of the metal atom plays an obvious role in altering the character of CH_2_O interaction with the nano-cages from pure ionic interaction into an ionic interaction with a partially covalent character. Additionally, the CH_2_O adsorption decreases the E_g_ value for Be_12_O_12_ and CuBe_12_O_12_ by 39.31% and 44.13%, respectively, and increases its value for KBe_12_O_12_ and MnBe_12_O_12_ by 87.74% and 43.42%, respectively.

It is found that the existence of an external static electric field (EF) can enhance or inhibit the adsorption energies of CH_2_O molecules on the Be_12_O_12_ as well as the MBe_12_O_12_ nano-cages, depending on the value and the orientation of the EF. Furthermore, the EF has an obvious influence on the E_g_ of CH_2_O/MBe_12_O_12_ complexes and, consequently, on their electrical conductivity. Thus, the electrical sensitivity of MBe_12_O_12_ nano-cages to CH_2_O gas can be controlled via EF. Moreover, the CH_2_O adsorption makes a red shift for the UV-vis spectrum of the Be_12_O_12_ and causes obvious changes in the absorption peaks of the MBe_12_O_12_ nano-cages in the visible region. Additionally, the EF has an obvious effect on the UV-vis spectra of CH_2_O/Be_12_O_12_ as well as the CH_2_O/MBe_12_O_12_ complexes.

Based on these results, the Be_12_O_12_ as well as the MBe_12_O_12_ nano-cages are candidate materials for removing and sensing the formaldehyde gas. The MBe_12_O_12_ nano-cages may be utilized as electrochemical and naked-eye sensors for CH_2_O gas.

## Figures and Tables

**Table 1:** Electronic properties of Be_12_O_12_ and MBe_12_O_12_ nano-cages (M = K, Mn, or Cu). HOMO and LUMO energy levels (eV), HOMO–LUMO gap (E_g_, eV), average binding energy per atom (E_b_, eV), NBO charges (Q, e), ionization potential (IP, eV), Fermi level (**E_F_**, eV), hardness (**η**, eV), electrophilicity (**ω**, eV), and dipole moment (D, Debye).

	Be_12_O_12_	KBe_12_O_12_	MnBe_12_O_12_	CuBe_12_O_12_
HOMO (α)	−8.463	−2.128	−2.281	−3.544
LUMO (α)	−0.253	−0.973	−0.547	−0.373
HOMO (β)	−8.463	−8.607	−3.803	−7.446
LUMO (β)	−0.253	−1.443	−1.224	−1.977
E_g_	8.210	0.685	1.057	1.568
E_b_	−5.630	−5.137	−5.201	−5.405
Q_Be_	1.210	1.241	1.199	1.185
Q_O_	−1.210	−1.249	−1.211	−1.194
Q_M_	-	0.096	0.237	0.108
IP	10.135	3.298	3.639	5.216
E_F_	−4.358	−1.786	−1.753	−2.760
η	4.105	0.343	0.529	0.784
ω	2.314	4.655	2.905	4.860
D	0.001	0.003	0.001	0.105

**Table 2:** The electronic configurations for M atom in a free state and MBe_12_O_12_ nano-cages.

Structure	4s	3d	4p
K	1.00	-	-
KBe_12_O_12_	0.46	0.23	0.41
Mn	2.00	5.00	-
MnBe_12_O_12_	0.63	5.76	0.41
Cu	1.00	10.0	-
CuBe_12_O_12_	0.76	9.67	0.48

**Table 3:** Adsorption properties of CH_2_O on MBe_12_O_12_ nano-cages. Adsorption energies (E_ads_, eV), HOMO and LUMO energy levels (eV), HOMO–LUMO gap (E_g_, eV), NBO charges (Q, e), and dipole moment (D, Debye).

	CH_2_O/Be_12_O_12_	CH_2_O/KBe_12_O_12_	CH_2_O/MnBe_12_O_12_	CH_2_O/CuBe_12_O_12_
E_ads_	−0.963	−2.518	−2.551	−1.541
HOMO	−7.869	−2.665	−3.007	−2.900
LUMO	−2.886	−1.380	−1.491	−2.024
E_g_	4.983	1.286	1.516	0.876
Q_Be1_	1.134	1.230	1.208	1.191
Q_O2_	−1.221	−1.233	−1.181	−1.177
Q_O3_	−1.210	−1.232	−1.191	−1.182
Q_O4_	−1.210	−1.229	−1.174	−1.181
Q_M_	-	0.443	0.570	0.578
Q_O1_	−0.516	−0.858	−0.853	−0.849
Q_H1_	0.194	0.134	0.134	0.134
Q_H2_	0.180	0.148	0.153	0.151
Q_C_	0.290	−0.128	−0.124	−0.126
$Q_{CH_{2}O}$	0.148	−0.703	−0.690	−0.690
D	5.387	8.494	6.804	6.962

**Table 4:** The electronic configurations for 2s and 2p orbitals for oxygen and carbon atoms of CH_2_O for free CH_2_O, CH_2_O/Be_12_O_12,_ and CH_2_O/MBe_12_O_12_.

Structure	O		C	
	2s	2p	2s	2p
CH_2_O	1.72	4.76	1.05	2.70
CH_2_O/Be_12_O_12_	1.66	4.84	1.07	2.62
CH_2_O/KBe_12_O_12_	1.66	5.18	1.07	3.04
CH_2_O/MnBe_12_O_12_	1.66	5.18	1.07	3.03
CH_2_O/CuBe_12_O_12_	1.66	5.18	1.07	3.03

**Table 5:** The estimated topological parameters. Electron densities (ρ), Laplacian of charge density ($\nabla^{2}\rho$), kinetic electron density (G(r)), potential energy density (V(r)), and energy density (H(r)). All units are in au.

Complex	BCP *	ρ	$\nabla^{2}\rho$	G(r)	V(r)	H(r)	−G(r)/V(r)
CH_2_O/Be_12_O_12_	Be1-O1	0.048	0.327	0.077	−0.072	0.005	1.069
	O2-H1	0.014	0.054	0.012	−0.010	0.002	1.200
CH_2_O/KBe_12_O_12_	Be1-O1	0.110	0.858	0.212	−0.211	−0.002	1.005
CH_2_O/MnBe_12_O_12_	Be1-O1	0.113	0.879	0.219	−0.219	−0.001	1.000
CH_2_O/CuBe_12_O_12_	Be1-O1	0.112	0.867	0.216	−0.215	−0.001	1.005

* The atom numbering shown in the Figure 6 is used.

## Data Availability

Data are contained within the article.

## Supplementary Materials

The following supporting information can be downloaded at: https://www.mdpi.com/article/10.3390/nano14010007/s1, Figure S1. Potential energy fluctuations during MD simulation as well as the atomic configuration after 500 fs at 300 K for (a) Be_12_O_12_, (b) KBe_12_O_12_, (c) MnBe_12_O_12_, and (d) CuBe_12_O_12_ nano-cages, Figure S2. FT-IR spectra for (a) Be_12_O_12_, (b) KBe_12_O_12_, (c) MnBe_12_O_12_, and (d) CuBe_12_O_12_ nano-cages, Figure S3. Non-optimized adsorption modes for CH_2_O on MBe_12_O_12_ nano-cage (M = K, Mn, and Cu). (a, b, and c) by O, H, and C atom of CH_2_O molecule on Be site of MBe_12_O_12_ nano-cage and (d, e, and f) by O, H, and C atom of CH_2_O molecule on O site of MBe_12_O_12_ nano-cage, respectively, Figure S4. Bond critical points of type (3,−1) for (a) CH_2_O/Be_12_O_12_, (b) CH_2_O/KBe_12_O_12_, (c) CH_2_O/MnBe_12_O_12_, and (d) CH_2_O/CuBe_12_O_12_ complexes.
