# Supplementary material for: M-Encapsulated Be12O12 Nano-Cage (M = K, Mn, or Cu) for CH2O Sensing Applications: A Theoretical Study
Source: Nanomaterials (Basel). 2023 Dec 19;14(1):7. doi: 10.3390/nano14010007 (PMC10780420; doi:10.3390/nano14010007)
Supplement: Supplementary file 1 [file nanomaterials-14-00007-s001.zip › nanomaterials-2754967-supplementary.pdf]

Article

M-Encapsulated $Be_{12}O_{12}$ Nano-Cage (M = K, Mn, or Cu) for CH_2O Sensing Applications: A Theoretical Study

Hatim Omar Al-Nadary [1], Khaled Mahmoud Eid [2], Heba Mohamed Badran [1,*] and Hussein Youssef Ammar [1,*]

[1] Physics Department, College of Science & Arts, Najran University, Najran 11001, Saudi Arabia; hoalnadary@nu.edu.sa

[2] Physics Department, Faculty of Education, Ain Shams University, Cairo 11566, Egypt; khaledmahmoud@edu.asu.edu.eg

* Correspondence: hmbadran@nu.edu.sa (H.M.B.); hyammar@nu.edu.sa (H.Y.A.)

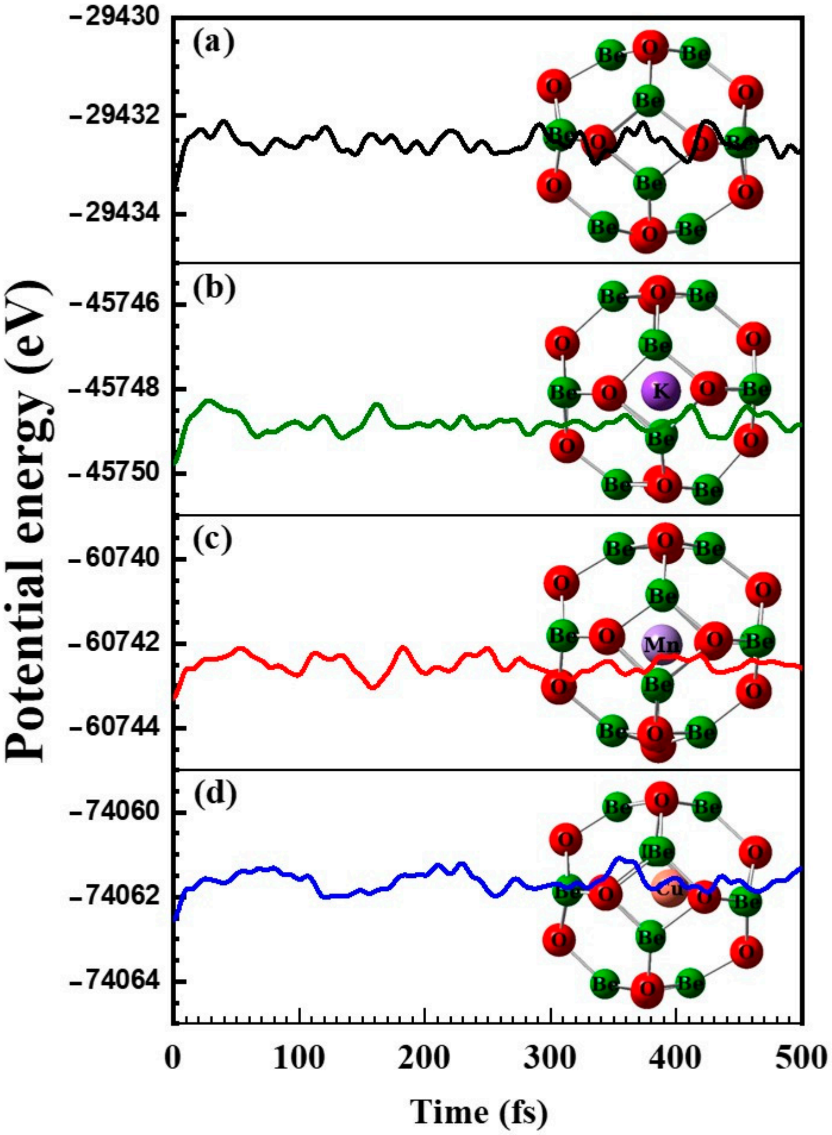

Figure S1. Potential energy fluctuations during MD simulation as well as the atomic configuration after 500 fs at 300 K for (**a**) $Be_{12}O_{12}$, (**b**) $KBe_{12}O_{12}$, (**c**) $MnBe_{12}O_{12}$, and (**d**) $CuBe_{12}O_{12}$ nano-cages.

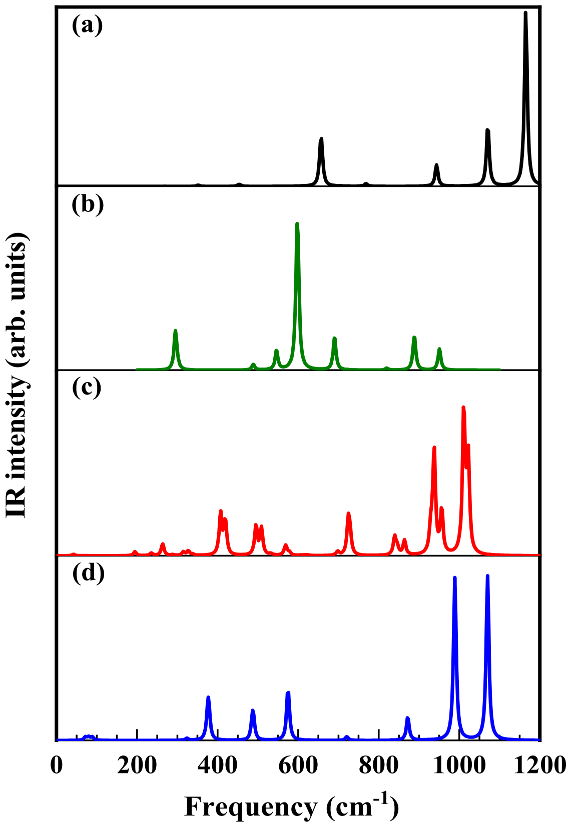

Figure S2. FT-IR spectra for (**a**) $Be_{12}O_{12}$, (**b**) $KBe_{12}O_{12}$, (**c**) $MnBe_{12}O_{12}$, and (**d**) $CuBe_{12}O_{12}$ nano-cages.

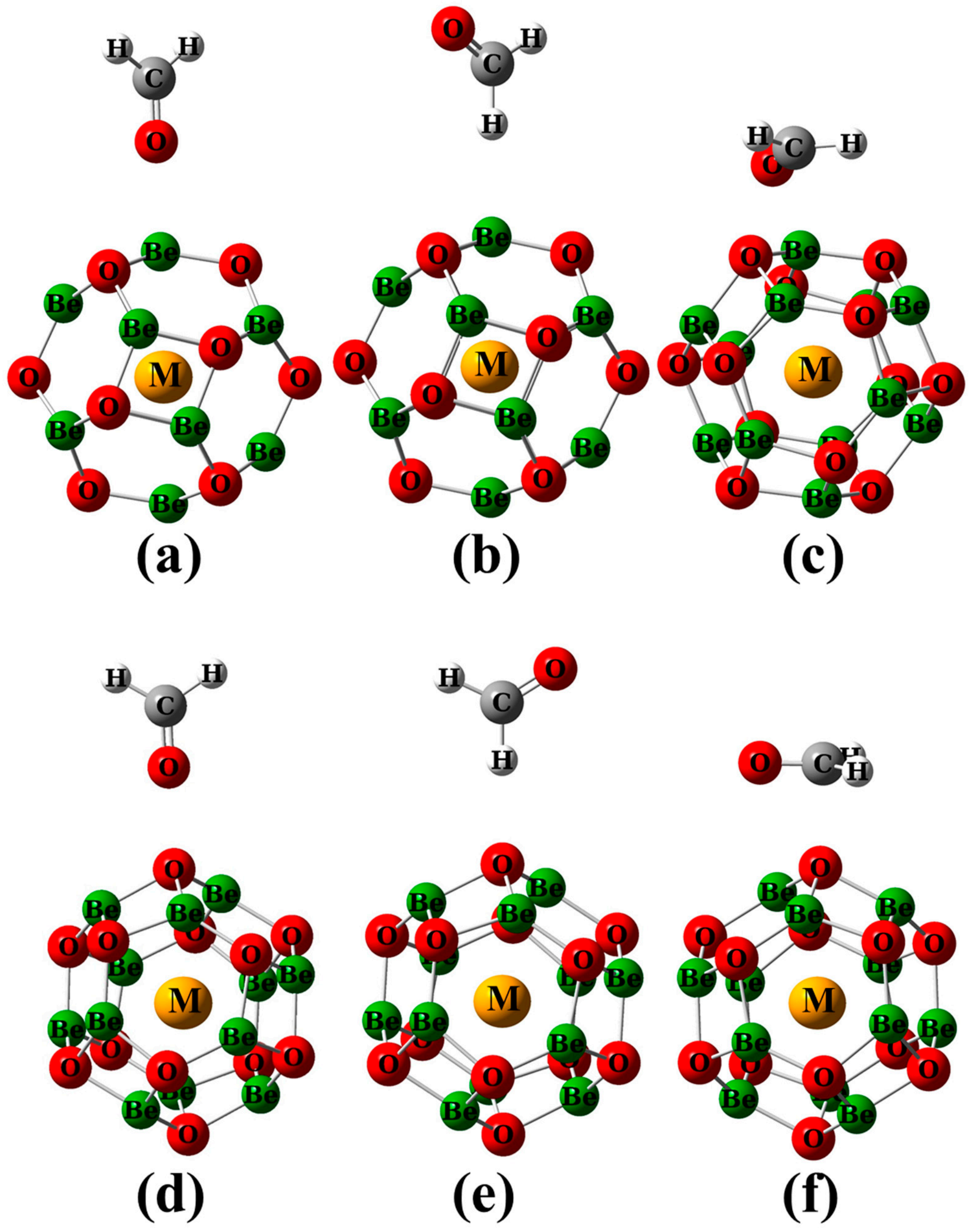

Figure S3. Non-optimized adsorption modes for CH_2O on $MBe_{12}O_{12}$ nano-cage (M = K, Mn, and Cu). (a, b, and c) by O, H, and C atom of CH_2O molecule on Be site of $MBe_{12}O_{12}$ nano-cage and (d, e, and f) by O, H, and C atom of CH_2O molecule on O site of $MBe_{12}O_{12}$ nano-cage, respectively.

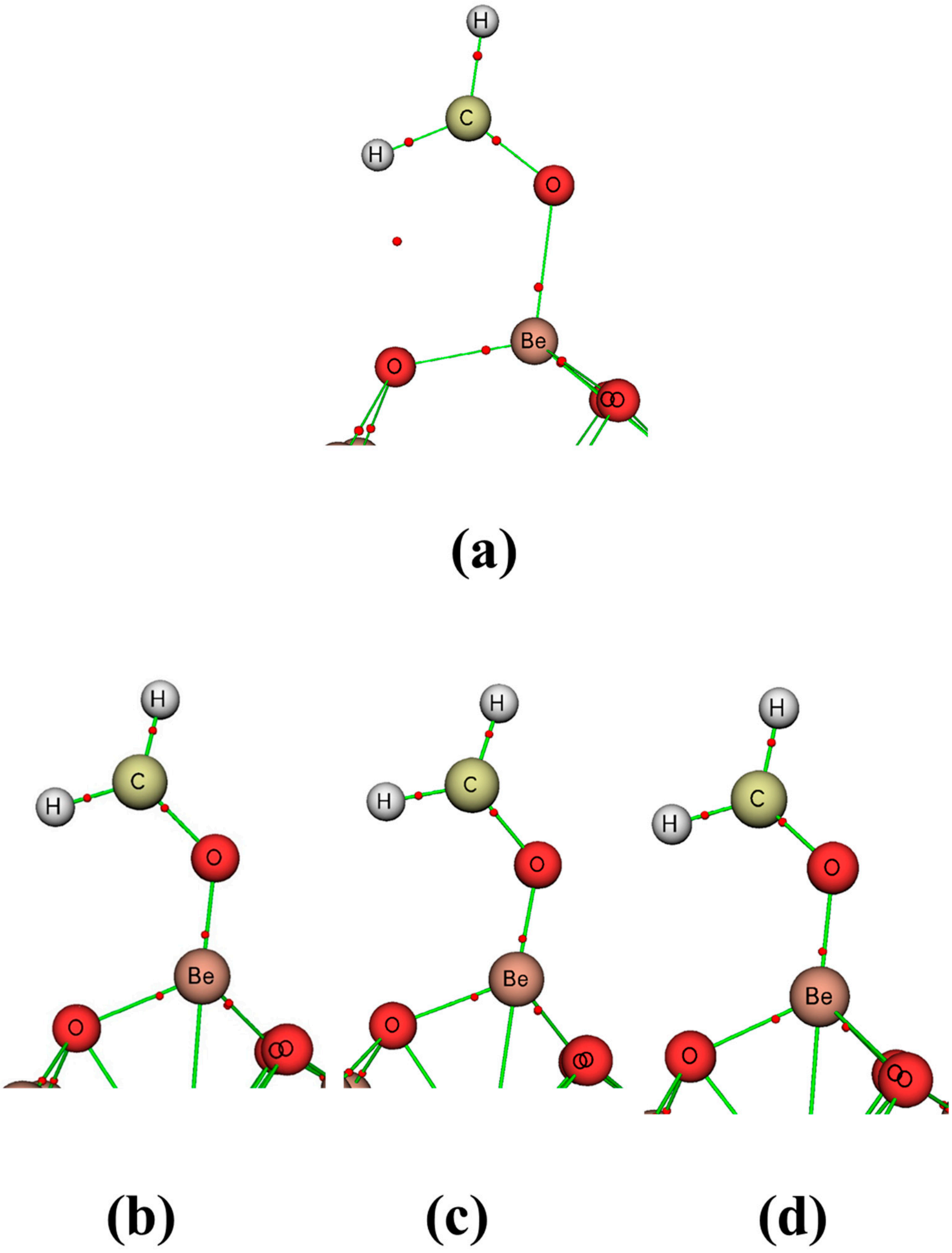

Figure S4. Bond critical points of type (3,−1) for (a) $CH_2O/Be_{12}O_{12}$, (b) $CH_2O/KBe_{12}O_{12}$, (c) $CH_2O/MnBe_{12}O_{12}$, and (d) $CH_2O/CuBe_{12}O_{12}$ complexes.
